# Prenatal exposure to lipopolysaccharide results in myocardial remodelling in adult murine offspring

**DOI:** 10.1186/1476-9255-10-35

**Published:** 2013-11-19

**Authors:** Yanling Wei, Wenhua Du, Xiuqin Xiong, Xiaoyan He, Ping Yi, Youcai Deng, Dongfeng Chen, Xiaohui Li

**Affiliations:** 1 The Institute of Materia Medica and Department of Pharmaceutics, College of Pharmacy, The Third Military Medical University, Chongqing, China; 2 Department of Gastroenterology, Research Institute of Surgery, Da ping Hospital, The Third Military Medical University, Chongqing, China; 3 Department of Ultrasound, Research Institute of Surgery, Da ping Hospital, The Third Military Medical University, Chongqing, China; 4 Department of Gynaecology, Research Institute of Surgery, Da ping Hospital, The Third Military Medical University, Chongqing, China

**Keywords:** Foetal development, Maternal inflammation, Myocardial remodelling, Inhibitor of NF-κB

## Abstract

**Background:**

The epigenetic plasticity hypothesis indicates that pregnancy exposure may result in adult-onset diseases, including hypertension, diabetes and cardiovascular disease, in offspring. In a previous study, we discovered that prenatal exposure to inflammatory stimulants, such as lipopolysaccharides (LPS), could lead to hypertension in adult rat offspring. In the present study, we further demonstrate that maternal inflammation induces cardiac hypertrophy and dysfunction via ectopic over-expression of nuclear transcription factor κB (NF- κB), and pyrrolidine dithiocarbamate (PDTC) can protect cardiac function by reducing maternal inflammation.

**Methods:**

Pregnant SD rats were randomly divided into three groups and intraperitoneally injected with a vehicle, LPS (0.79 mg/kg), or LPS (0.79 mg/kg) plus PDTC (100 mg/kg) at 8 to 12 days of gestation. The offspring were raised until 4 and 8 months old, at which point an echocardiographic study was performed. The left ventricular (LV) mass index and apoptosis were examined.

**Results:**

At 4 months of age, the LPS offspring exhibited augmented posterior wall thickness. These rats displayed left ventricle (LV) hypertrophy and LV diastolic dysfunction as well as a higher apoptotic index, a higher level of Bax and a lower level of Bcl-2 at 8 months of age. The protein levels of NF-κB (p65) in the myocardium of the offspring were measured at this time. NF-κB protein levels were higher in the myocardium of LPS offspring. The offspring that were prenatally treated with PDTC displayed improved signs of blood pressure (BP) and LV hypertrophy.

**Conclusions:**

Maternal inflammation can induce cardiac hypertrophy in offspring during aging accompanied with hypertension emergence and can be rescued by the maternal administration of PDTC (the inhibitor of NF-κB).

## Background

Immunological and inflammatory responses are believed to play major roles in the pathophysiology of cardiovascular disease (CVD). However, the detailed mechanism is not yet clear. Myocardial remodelling, the major risk factor underlying cardiovascular morbidity and mortality, is commonly observed in individuals with essential hypertension. Myocardial remodelling is primarily a disease of the elderly [1]. It involves a continuum of changes in the structure and function of the myocardium that usually occur in cardiac hypertrophy (CH) and heart failure (HF) as a result of pathological processes [2]. Chronic hypertension, congenital heart disease with intracardiac shunting, and cardiac valvular disease may also lead to heart remodelling [3,4]. However, we identified a new agent in addition to common injury that could result in myocardial remodelling. This agent is developmental programming that may be affected by maternal inflammation. It would be valuable to investigate the adverse events of this agent during early life and adult-onset diseases.

The perturbations during gestation and neonatal life can influence long-term metabolic homeostasis factors, such as weight, food intake, serum lipids, and insulin resistance [5]. Elevated blood pressure in offspring has also been studied, which may be triggered by the antenatal administration of glucocorticoids, hypoxia, impaired placental perfusion, gestational diabetes, neonatal hyperoxia, or the modifications of nutritional regimens [6]. Systemic inflammatory response during pregnancy also represents one type of stressful event that can affect the foetus, and its effects on developmental programming are not clear [7]. Recently, we have found that prenatal exposure to lipopolysaccharide (LPS), which is commonly used to mimic prenatal infection, could result in increased TNF-α and IL-6 levels in pregnant rats and induce hypertension in adult offspring [8]. These effects indicate that maternal inflammation has effects on foetal developmental programming that could induce offspring adulthood disease. Furthermore, maternal immunological and inflammatory responses may have long-term effects on tissue structures and functions in offspring, with the heart and blood vessels being particularly affected. Therefore, in this study, we investigated how cardiac function and structure are influenced by maternal inflammation.

Lipopolysaccharides are the major constituents of the outer membrane of Gram-negative bacteria. They act as endotoxins, which are nonspecific immunostimulants, to mimic the bacterial inflammatory response [9]. LPS initiates a series of phosphorylation events by binding to Toll-like receptor 4 (TLR4) and promoting the translocation of the nuclear transcription factor (NF)-κB into the nucleus [10]. NF-κB is a major mediator of LPS signalling and is a central regulator in immunological and inflammatory processes [11,12]. Pyrrolidine dithiocarbamate (PDTC) is a pharmacological inhibitor of NF-κB [13]. Various studies have shown that PDTC can prevent hypertension in spontaneously hypertensive rats and excessive ECM deposition in maladaptive cardiac remodelling during HF in rats [13,14].

In this study, we determined that prenatal exposure to inflammatory stimulants could result in myocardial remodelling with hypertension in adult offspring due to ectopic over-expression of NF-κB, and we uncovered the therapeutic benefits of maternal PDTC treatment in cardiac remodelling and hypertension caused by maternal inflammation.

## Methods

### Animals

This study was conducted in accordance with the Guide for the Care and Use of Laboratory Animals published by the US National Institutes of Health (NIH Publication No. 85–23, revised 1996) and was approved by the local animal ethics committee at the Third Military Medical University. Animals that were previously reported to become hypertensive after prenatal treatment with LPS early in gestation were used in this study [8]. Briefly, female and male Sprague Dawley (SD) rats that were purchased from the Animal Center of the Third Military Medical University (Chongqing, China) were mated (one female to one male; mating was confirmed based on the analysis of the vaginal plug and vaginal smear).

### Dams and litters

Throughout pregnancy, the rats were housed in standard rat cages with ad libitum access to water and food (standard lab rat chow). One week after they were acclimatised in our institute, the dams were randomly divided into the 3 following groups (n = 8 in each group): the control group, the LPS group and the LPS + PDTC group. The pregnant rats in the control group were intraperitoneally (i.p.) administered the vehicle daily from days 8 to 14 of gestation. In the LPS group, the rats were administered i.p. 0.79 mg/kg LPS (Escherichia coli 026:B6, Sigma, St. Louis, MO, USA) on days 8, 10 and 12. The rats in the LPS + PDTC group were administered i.p. 0.79 mg/kg LPS plus 100 mg/kg PDTC (Sigma Chemical) on days 8, 10 and 12. Gestation lasted for 20–22 d. All offspring rats from the same group were mixed together; they were redistributed within the same group so that there were 4 male and 4 female offspring rats per lactating mother. In each group, one lactating mother received the remaining litters after redistributed no matter how many offspring rats were left. The rats were left undisturbed until they reached 4 weeks of age, when they were weaned. At that time, all of the offspring rats from the same group were mixed together and were randomly allocated into cages. The offspring rats lived for 4 or 8 months until they were considered to be young adults and aged adults, respectively. Both male and female offspring rats were studied, and there were no gender differences in the current study.

### Blood pressure measurement

Systolic blood pressure (SBP) was measured in six conscious offspring rats from each group at 2, 4, 6 and 8 months of age using the standard tail-cuff method (ML125; Powerlab, ADInstruments, Castle Hill, NSW, Australia) [7]. The rats were initially trained to adapt to the method before the valid measurements were taken at least three times. Before SBP measurements, the rats were placed inside a warming chamber (approximately 34°C) for 15 min and were then placed in plastic restraints. A cuff with a pneumatic pulse sensor was attached to the tail. For each rat, the mean SBP was calculated from three consecutive recordings. The mean of all of the obtained values was used in the calculations.

### Echocardiographic evaluation

High-resolution echocardiography has been shown to be an effective tool for the in vivo study of cardiovascular function and structure in small rodents [15,16]. At 4 or 8 months of age, transthoracic echocardiography was performed on the offspring rats after they were anaesthetised with pentobarbital sodium (35 mg/kg, i.p.) [17-19]. The chest of each rat was shaved, and a layer of acoustic-coupling gel was applied to the thorax. Two-dimensional (2D) echocardiography was performed using a commercially available 12-MHz linear-array transducer system (IE33-S12-MHz; Philips, Hamburg, Germany). The rats were placed in the left lateral decubitus position. M-mode recordings of the LV were obtained at the level of the mitral valve in the parasternal view using 2D echocardiographic guidance in both the short- and long-axis views. Pulsed-wave Doppler was used to examine the mitral diastolic inflow from the apical four-chamber view. The 2D echocardiographic measurements included the LV end-systolic and end-diastolic diameters (LVESD and LVEDD, respectively), end-diastolic LV posterior wall thickness (PWT), thickness of the interventricular septum (IVST), ejection fraction (EF) and fractional shortening (FS). FS was calculated as (LVEDD–LVESD)/LVEDD. All of the measurements were made in a blinded fashion from digital images that were captured using analysis software that was installed on the echocardiographic machine. For each measurement, the data from three consecutive cardiac cycles were averaged. The 2D and Doppler images were obtained at a speed of 100 mm/s.

The diameters of the aortic root and left atrium were obtained using M-mode with the scan head on the long-axis and the beam parallel to the axis of the aortic valve. The LV systolic function was evaluated by estimating the EF, shortening fraction and cardiac output from the images that were obtained in M-mode of the LV short-axis. The ventricular diastolic function was assessed by describing the transmitted Doppler signal. The E and A wave velocities and gradients were measured in a modified parasternal long-axis with the beam placed on the tip of the mitral leaflet. The E/A index, mitral deceleration time, and myocardial performance index (Tei index) were calculated [20].

### Left ventricular hypertrophy index

After echocardiography, the rats were sacrificed by decapitation, and the chest cavity was opened immediately. The hearts were excised rapidly and placed into ice-cold saline to remove the blood. The hearts then were weighed. The atria were removed from the harvested hearts, and the left ventricles were separated. The interventricular septum remained as a part of the left ventricle [21,22]. The heart index (HI) and LV mass index (LVMI) were calculated as the ratio of heart weight (in mg) and LV wet weight (in mg) to body weight (in g), respectively.

### Histological examination

The LV tissue was sliced into 3-mm sections, which were fixed in 10% neutral-buffered formalin. The tissues were embedded in paraffin, sectioned at 5 μm and stained with hematoxylin-eosin (HE). All of the slices were assessed using a computer-assisted colour image analysis system (Motic Images Advanced 3.0, GENEQ, Montreal, Canada). The transverse diameter (TDM) and cross-sectional area (CSA) of the cardiac myocytes were determined in the HE-stained slices. For the measurement of the single-cardiomyocyte cross-sectional area and width, a total of 30 left ventricular myocytes were sectioned transversely at the level of the nucleus and randomly chosen from each section at 400× magnification and traced.

### Preparation of nuclear protein extract and western blot analysis

At 8 months of age, six offspring rats from each group were anaesthetised with pentobarbital i.p. and decapitated. The hearts were removed immediately and placed in ice-cold NaCl/Pi before they were cut into pieces. The nuclear protein extracts were prepared according to the instructions that were provided with the NE-PER Nuclear and Cytoplasmic Extraction Reagents kit (Pierce Biotechnology, Rockford, IL, USA). Following protein quantification, the nuclear proteins (50 μg) were electrophoresed on a 10% sodium dodecyl sulphate–polyacrylamide gel, transferred to nitrocellulose, immunoblotted with an anti-NF-κB (p65) antibody (1:1000 dilution; Santa Cruz Biotechnology, Santa Cruz, CA, USA), according to the manufacturer’s instructions, and visualised with peroxidase and an enhanced chemiluminescence system (ECL Kit; Pierce Biotechnology Inc., Rockford, IL, USA).

### Measurement of myocyte apoptosis by TUNEL assay

The apoptotic cardiac cells were identified using the terminal deoxynucleotidyl transferase-mediated dUTP nick end labelling (TUNEL) assay in 5-μm thick, formalin-fixed, paraffin-embedded sections [23,24]. The rat myocardial tissue sections were obtained from the 3 groups of offspring at 8 months of age. The TUNEL assay (Roche, Germany) was performed according to the manufacturer’s protocol and counterstained with DAPI to mark all nuclei. The slides were examined microscopically at 400× magnification. Under fluorescent light with 490 nm, TUNEL-positive cells were counted per 10^6^ cardiomyocyte nuclei. The apoptotic index was calculated as the number of apoptotic cardiomyocyte nuclei per total cardiomyocyte nuclei.

### Western blotting

The LV were removed at the end of the experiment, and they were lysed with lysis buffer. After sonication, the lysates were centrifuged, and the proteins were separated by electrophoresis (SDS-PAGE (10–14%)) and transferred onto a polyvinylidene difluoride (PVDF)-plus membrane. They were probed with a rat polyclonal Bax antibody (sc-483, 1:500, Santa Cruz Biotechnology Inc., CA, USA) and a mouse monoclonal Bcl-2 antibody (sc-7382, 1:500, Santa Cruz Biotechnology Inc., CA, USA). The peroxidase activity was detected using the Pierce ECL Western Blotting Substrate. For the densitometric analysis, the immunoreactive bands were scanned with a Luminescent Imaging Analyzer (LAS-4000, Fuji, Japan) and were quantified using the Multi Gauge software (Fuji, Japan). A monoclonal antibody against β-actin (sc-47778, 1:1000, Santa Cruz Biotechnology Inc., CA, USA) was used as an internal control.

### Statistics

All of the data are expressed as the mean ± SEM and were analysed using the SPSS 16.0 software package (SPSS, Chicago, IL, USA). Comparisons between the groups were made using a one-way ANOVA with Fisher’s least significant difference (LSD) post hoc test. *P* < 0.05 was considered to be statistically significant.

## Results

### Mother rats and offspring rats

There was no significant difference in the mean number of progeny rats among the 3 groups. Additionally, the ratio of male births to female births in each group and the body weights of the newborn offspring rats showed no differences among the 3 groups.

### SBP in adult offspring

**Figure 1:**
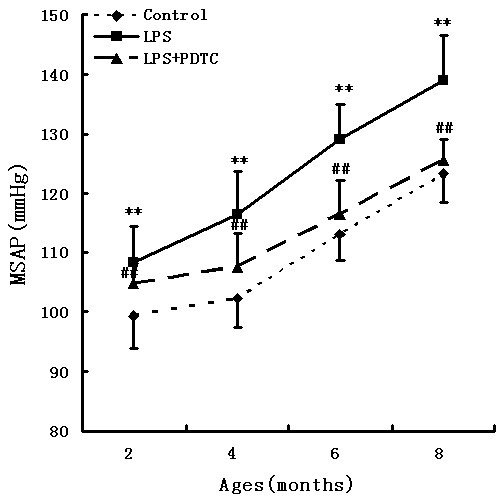
**Prenatal exposure to LPS influences SBP in rat offspring.** Pregnant rats were randomly divided into three groups (n = 8 in each group): control, LPS and LPS + PDTC. SBP in the offspring was measured using the standard tail-cuff method. **P < 0.05 compared with the controls; ^##^P < 0.05 compared with the offspring of the LPS-treated rats. There was no significant difference between the control group and the LPS + PDTC group (one-way analysis of variance).

The SBP levels were significantly increased in the LPS group compared with the control and LPS + PDTC groups during 6 and 8 months of age. At 6 months of age, SBP in the LPS-treated offspring rats reached the standard level of hypertension. At 8 months of age, the LPS group reached 138.85 ± 7.68 mmHg, which was significantly higher compared with that in the control (123.64 ± 4.68 mmHg) and LPS + PDTC (125.54 ± 3.54 mmHg) groups. There was no significant difference between the control group and the LPS + PDTC group (Figure 1).

### Effects of prenatal LPS exposure on left-ventricular morphometry and function

There were no significant differences in the heart rates between the three groups. As indicated in Table 1, at the end of 4 months, the PWTs were significantly increased in the adult offspring in the LPS-treated group compared with those in the control and LPS + PDTC groups (P < 0.05). However, there were no differences in the LV systolic and diastolic functions among the three groups. The 8-month-old offspring from both sexes of the LPS group indicated echocardiographic signs of increased PWT thickness and IVST and LV diastolic dysfunction, which included a decreased mitral A-wave maximum velocity, isovolumetric relaxation time, and deceleration time of the mitral valve as well as increased E/A and Tei indexes (Table 1). There were no significant differences of these parameters between the LPS + PDTC group and the control group.

**Table 1:** Comparison of LV remodelling and function before (4 months) and after (8 months) hypertension

	**Age (4 months)**			**Age (8 months)**		
	**Control**	**LPS**	**LPS + PDTC**	**Control**	**LPS**	**LPS + PDTC**
HR (bpm)	336 ± 12	340 ± 27	338 ± 36	308 ± 5	302 ± 7	306 ± 9
LVEDD (mm)	7.47 ± 0.25	7.78 ± 0.30	7.44 ± 0.28	7.83 ± 0.36	7.94 ± 0.56	7.79 ± 0.35
LVESD (mm)	4.11 ± 0.22	3.81 ± 0.12	4.08 ± 0.29	4.27 ± 0.30	3.78 ± 0.28*	4.13 ± 0.15^#^
PWT (mm)	2.02 ± 0.15	2.49 ± 0.23*	1.99 ± 0.11^#^	2.42 ± 0.07	2.57 ± 0.10*	2.45 ± 0.27^#^
IVST (mm)	1.88 ± 0.18	1.66 ± 0.27	1.81 ± 0.17	2.01 ± 0.04	2.20 ± 0.10*	2.02 ± 0.03^#^
Mitral E maximum						
vel. (mm/s)	1130 ± 46	1267 ± 192	1283 ± 303	1281 ± 105	1305 ± 79	1291 ± 124
Mitral A maximum						
vel. (mm/s)	691 ± 17	693 ± 46	671 ± 31	908 ± 63	572 ± 43*	916 ± 57^#^
Mitral E/A index	1.63 ± 0.05	1.83 ± 0.23	1.93 ± 0.54	1.41 ± 0.14	2.28 ± 0.15*	1.41 ± 0.14^#^
Mitral deceleration time (ms)	48.65 ± 2.97	44.67 ± 6.28	49.45 ± 4.95	42.05 ± 3.80	32.12 ± 3.17*	41.43 ± 2.67^#^
Myocardial performance (Tei) index	0.35 ± 0.02	0.42 ± 0.03	0.39 ± 0.01	0.34 ± 0.02	0.48 ± 0.03*	0.34 ± 0.02^#^
**Parasternal short-axis**						
FS (%)	45 ± 4.7	51 ± 1.6	45 ± 5.8	45 ± 5.1	48 ± 2.0	47 ± 4.3
EF (%)	75 ± 5.2	80 ± 4.7	74 ± 6.3	75 ± 5.4	78 ± 1.8	77 ± 4.4
**Parasternal long-axis apical**						
Ao end-systolic diameter (mm)	3.54 ± 0.28	3.40 ± 0.66	3.56 ± 0.14	3.75 ± 0.12	3.91 ± 0.13	3.80 ± 0.12
Vp (cm/s)	1322 ± 88.6	1233 ± 109.1	1354 ± 154.6	1160 ± 117.1	1040 ± 180.3	1146 ± 101.3
VTI (mmHg)	8.22 ± 0.15	8.53 ± 0.32	8.35 ± 0.33	7.76 ± 0.54	7.88 ± 0.68	7.64 ± 0.59

A summary of the most relevant cardiac measurements that were obtained at 4 and 8 months of age using echocardiography. *HR*, Heart rate; *LV*, Left ventricle; *LVEDD*, LV end-diastolic diameters; *LVESD*, LV end-systolic diameters; *PWT*, End-diastolic LV posterior wall thickness; *IVST*, The thickness of the interventricular septum; *Ao*, Aorta; vel, velocity; *EF*, LV ejection fraction; *FS*, LV fractional shortening; *Vp*, Ao peak ejection vel.; and *VTI*, Ao outflow vel./time integral.

The data are presented as the mean ± SEM (n = 6 in each group). *P < 0.05 vs. control group; ^#^P <0.05 vs. LPS group.

### Effect of prenatal exposure to LPS on the LV hypertrophy index of adult offspring

**Figure 2:**
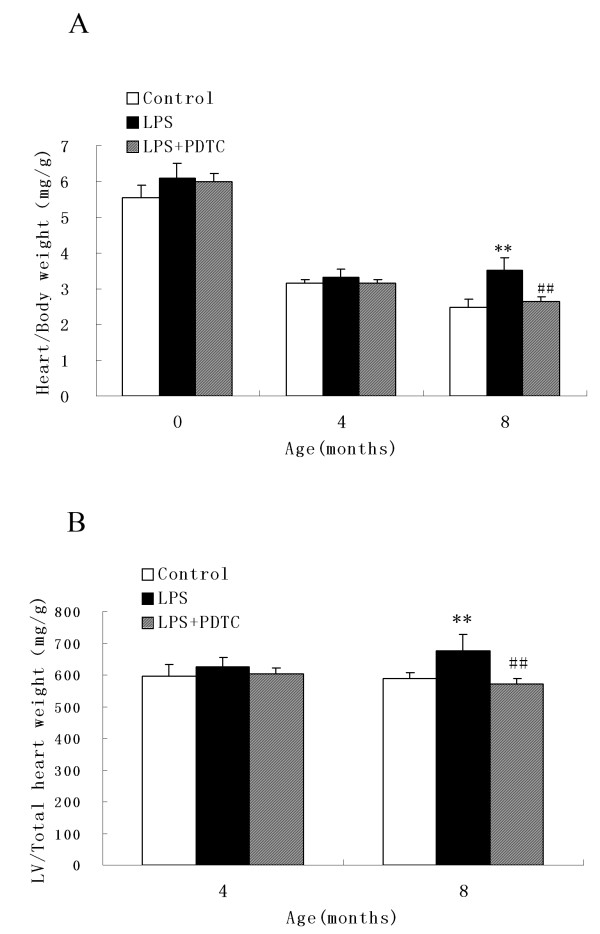
**Effect of prenatal exposure to LPS on the heart/body weight ratio and left ventricle (LV)/total heart weight.** The heart/body weight ratio **(A)** and left ventricle (LV)/total heart weight **(B)** in the offspring from the LPS or LPS + PDTC groups. **P < 0.05 compared with the controls; ^##^P <0.05 compared with the offspring of the LPS-treated rats. There was no significant difference between the control group and the LPS + PDTC group (one-way analysis of variance).

Compared with the control rats, the heart weight, LV weight, HI and LVMI were markedly increased in the LPS-treated group at the end of 8 months (P < 0.05). There were no differences in the HI and LVMI among the three groups at the end of 4 months. All of the indices significantly decreased in the LPS + PDTC-treated group compared with those in the LPS-treated group (P < 0.05) (Figure 2A and B).

### Histological examination

**Figure 3:**
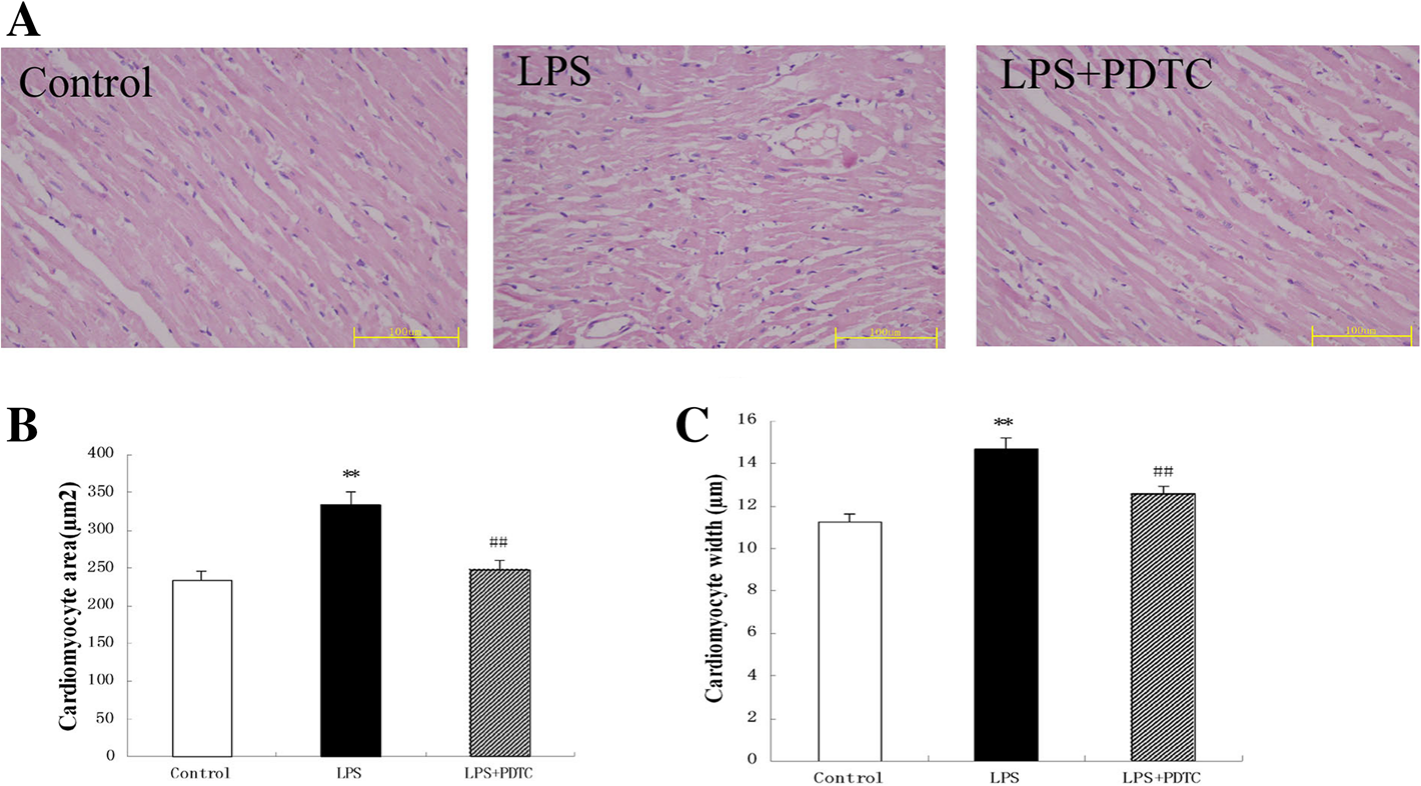
**Effect of prenatal exposure to LPS on cardiac myocyte hypertrophy in adult offspring. (A)** Photomicrographs showing the typical myocardial structure in the various groups (hematoxylin-eosin stain; 400×). **(B)** Quantitative morphometric analysis of the cardiomyocyte area for a single cell. **(C)** Quantitative morphometric analysis of the cardiomyocyte width for a single cell. **P < 0.05 compared with the controls; ^##^P <0.05 compared with the offspring of the LPS-treated rats.

Using an optical microscope, we observed that the myofibrillae were aligned without disruption in the control rats, and the morphology and structure of the nuclei and cells were normal. In contrast, the cardiomyocytes were hyperplastic and hypertrophied in the LPS-treated group at the end of 8 months. Following the treatment with LPS + PDTC, the morphology of the myocardium improved significantly (Figure 3). We also examined the cardiomyocyte size in the left ventricle and observed an increase in the cross-sectional myocyte area and width in the LPS-treated rats compared with the control rats. This increase was significantly prevented by treatment with the prenatal injection of PDTC.

### Prenatal exposure to LPS increases the NF-κB (p65) content in the myocardium

**Figure 4:**
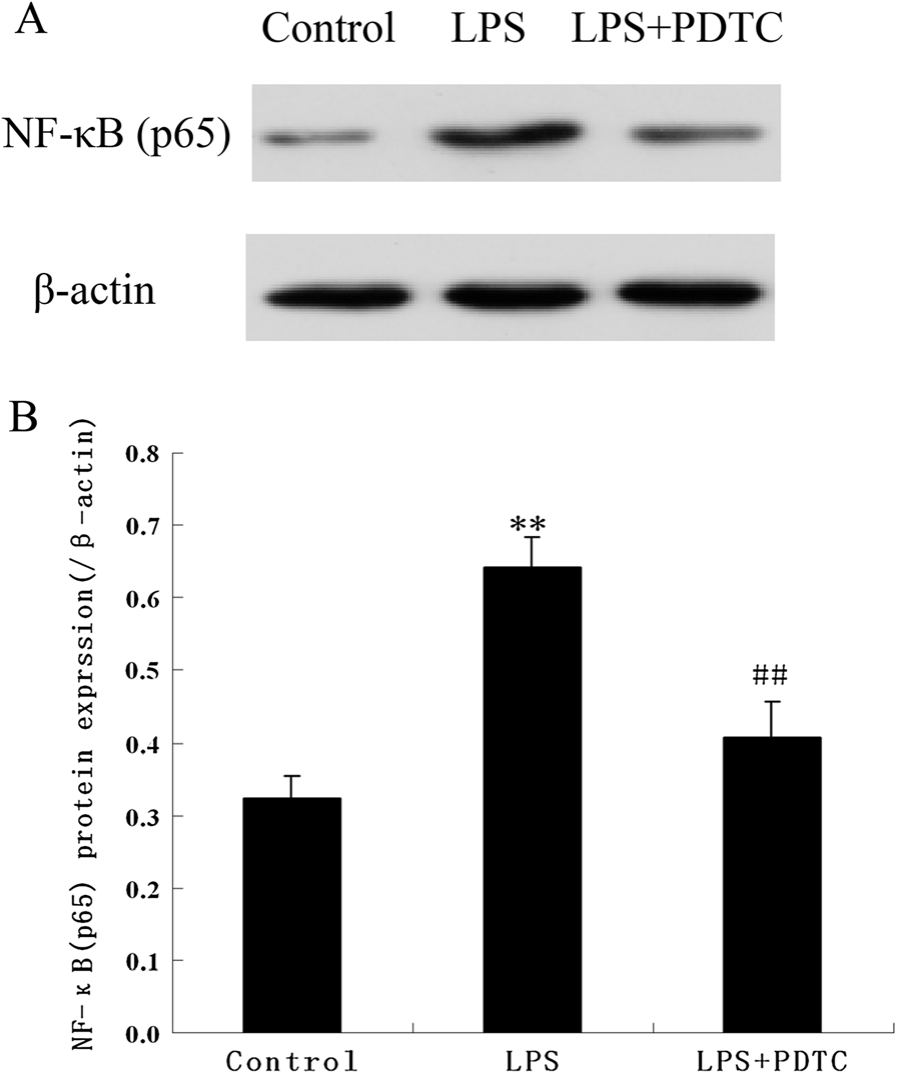
**The pictures (A) show the WB results of activation of the (NF)-κB pathway in offspring from the control, LPS and LPS + PDTC groups.** The graphs **(B)** show the results of the densitometric analysis. Similar results were obtained forix animals in each group. The data are the mean ± SEM. **P < 0.05 compared with the controls; ^##^P < 0.05 compared with the offspring of the LPS-treated rats.

At 8 months of age, the rats were sacrificed, and nuclear extracts were prepared from the heart to investigate NF-κB (p65) expression by western blotting. As shown in Figure 4A and B, the expression of p65 in the LPS-treated group was significantly higher compared to that in the control and LPS + PDTC groups. This result indicates that prenatal exposure to inflammatory stimuli can activate the NF-κB signalling cascade in offspring myocardium.

### Apoptosis

**Figure 5:**
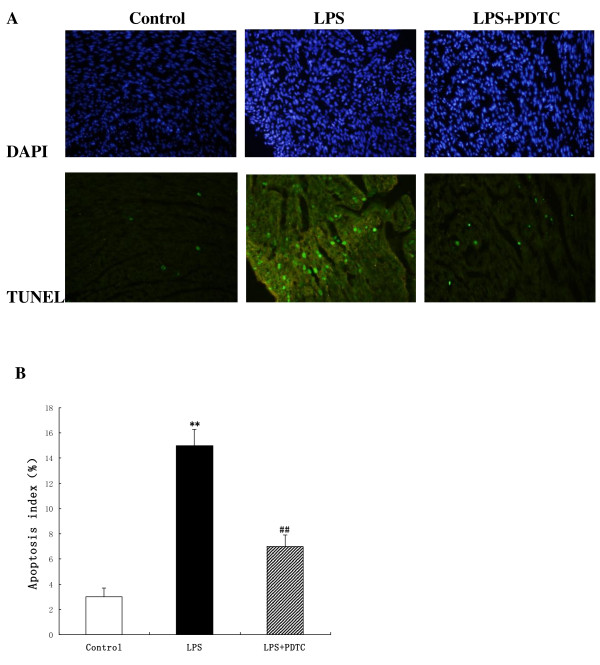
**TUNEL analysis in control, LPS and LPS + PDTC groups. (A)** Nuclei are shown in blue after staining with DAPI. TUNEL-stained cells show green fluorescence in cells with single-stranded DNA breaks. **(B)** The apoptotic index analysis of the cardiomyocytes in the heart. **P < 0.05 vs. control group; ^##^P < 0.05 vs. LPS group.

Apoptotic cardiomyocytes were detected by TUNEL staining in the hearts. Apoptotic nuclei were stained brown. The apoptotic index in the LPS-treated animals was significantly increased compared with that in the control and LPS + PDTC groups (Figure 5).

### Myocardial expression of Bcl-2 and Bax

**Figure 6:**
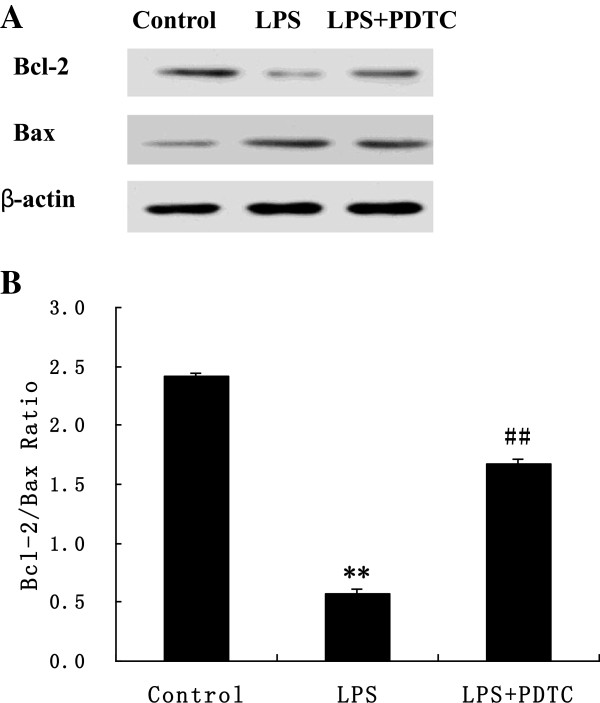
**The pictures (A) show the results of WB results on Bcl-2 and Bax.** The graphs **(B)** show the quantitative analysis on the Bcl-2/Bax ratio by western blotting. Lane 1 represents the control tissue, lane 2 represents the LPS-treated tissue, and lane 3 represents the LPS + PDTC-treated tissue. Each value represents the mean ± SEM of six rats. **P < 0.05 vs. control group; ^##^P < 0.05 vs. LPS group.

As shown in Figure 6, prenatal exposure to LPS decreased the expression of the Bcl-2 protein and increased the expression of the Bax protein in the myocardium, which resulted in a decrease of the Bcl-2/Bax ratio; this result indicates that the prenatal exposure to LPS induced apoptosis in the offspring rats. Furthermore, the intraperitoneal administration of PDTC prevented the decrease of the Bcl-2/Bax ratio.

## Discussion

A number of studies suggested that the development of CVD may be related to early events in childhood [25,26]. The study of the long-term effects of certain prenatal insults on cardiac structure and function has become increasingly relevant [27]. Previous studies have shown that changes in the intrauterine environment (e.g., the restriction of maternal protein and calories or prenatal exposure to glucocorticoid, alcohol or IL-6) trigger early programming, which results in the development of type 2 diabetes mellitus, hypertension, dyslipidaemia and CVD in adult offspring [28-32]. In our previous study, we found that prenatal exposure to LPS could result in increases of blood pressure and body weight in rats [7].

In the present study, we explored the effects of maternal LPS exposure in rats during gestation and its consequences for BP and myocardial remodelling in adult life. The MSAP of the offspring in the LPS-treated group started to increase beginning at 2 months of age. The rats that were exposed prenatally to LPS gradually developed hypertension at 6 months of age [33]. The blood pressure eached 138.85 ± 7.68 mmHg at the end of 8 months of age. A similar increase in the MSAP was not observed in either the control or LPS + PDTC-treated groups.

In the present study, we tried to analyse the presence of different patterns of remodelling in the hearts of adult rats that were prenatally exposed to LPS and to determine their functional implications. In a clinical setting, patients usually display different geometric patterns of myocardial remodelling. Using the mass index and the LV relative wall thickness as echocardiographic variables, the patients are usually divided into four different geometric patterns: normal (normal mass index and normal relative thickness), concentric remodelling (normal mass index and increased relative thickness), concentric hypertrophy (increased mass index and increased relative thickness), and eccentric hypertrophy (increased mass index and normal relative thickness). The remodelling pattern is a predictor of cardiovascular events [34]. In the present study, we found that at the end of 4 months, when the rats were defined as young adults, LPS offspring hearts showed increased PWT thickness but normal mass index with hypertension formation. Concentric remodelling fit this pattern. At the end of 8 months and with blood pressure raised in the LPS group, an increased mass index and increased PWT and IVST thicknesses were observed. Concentric hypertrophy occurred in the LPS group. Furthermore, hyperplastic and hypertrophic cardiomyocytes were also observed in the LPS-treated group. Systolic function, which was determined by echocardiography, was not changed among the groups. In contrast, the E/A and Tei indexes in the LPS group were significantly increased compared with that in the control and LPS + PDTC groups, which indicates moderate diastolic dysfunction in the LPS group. In this study, it is clear that the adult offspring developed cardiomyocyte hypertrophy with hypertension. It is likely that the cardiac remodelling was likely programmed by the adverse intrauterine environment that was caused by maternal inflammation. The present findings suggested that body and cardiac growth are influenced by prenatal inflammatory exposure conditions. These relationships between organ size and function underscore one of the basic biological compensatory properties: the inherent ability to increase or decrease mass (hypertrophy or atrophy) and to alter tissue configuration in a manner that is directly related to functional requirements. This study may represent a novel agent that can induce myocardial remodelling, may might also be an influential factor that contributes to increased CVD morbidity and mortality.

NF-κB is a key transcription factor that is involved in the regulation of many genes; it especially regulates inflammation-associated immediate-early genes, and it is closely associated with cardiovascular diseases, such as hypertension, atherosclerosis, ischaemia reperfusion injury and heart failure [13]. When a cell is quiescent, NF-κB (p65) is predominantly restricted to the cytoplasm. Upon stimulation (for example, by cytokines or in response to inflammation), NF-κB is activated and translocated from the cytoplasm into the nucleus, where it initiates the genetic transcription of factors, such as TNF-α and IL-6 [35]. Previous studies by us and others have demonstrated that LPS can induce an inflammatory response to infectious bacteria in a pregnant mother that could be abolished by PDTC treatment [7,9]. In the current study, we further demonstrated that the LPS-induced abnormalities of cardiac structure and function could be counteracted by the inhibition of the NF-κB cascade with PDTC. These results indicate that an intense inflammatory response occurred in pregnant rats following LPS exposure, and this response could induce cardiovascular disease, such as myocardial remodelling and hypertension. The NF-κB signalling pathway is most likely involved in this programming. Furthermore, in 8-month-old offspring rats from the LPS-treated group, NF-κB was primarily localised in the nucleus of cardiomyocytes. This phenomenon indicates the high sensitivity of the LPS-treated group to environmental stress compared with the control group. However, PDTC could reverse the LPS-mediated LV hypertension and myocardial remodelling in adult offspring rats by inhibiting the NF-κB pathway, which can drive maternal inflammatory reactivity.

It is noteworthy, however, that the mechanisms of the myocardial remodelling induced by maternal LPS exposure remain unclear. The exact mechanisms that are responsible for the maternal inflammation-induced myocardial remodelling in offspring also remain unclear. To investigate possible mechanisms that may contribute to prenatal LPS-induced offspring myocardial hypertrophy, myocardial apoptosis in offspring rats was evaluated in the present study. Previous studies demonstrated that apoptosis is involved in the cardiac remodelling and substantial loss of cardiomyocytes in genetic hypertension [36] and in renovascular hypertension [37]. Cardiomyocyte apoptosis enhances the cardiac remodelling of hypertensive rats and affects the cardiac interstitium [38]. Cardiomyocyte apoptosis induced by prenatal exposure to LPS may cause myocardial remodelling in the current study. Ventricular cardiomyocytes are normally considered to be terminally differentiated cells, and the capacity for cardiac repair by cardiomyocyte regeneration may be significantly decreased shortly after birth. The previous study demonstrated that cardiomyocyte growth under physiological and pathological conditions is believed to be restricted to cellular hypertrophy [39]. However, a recent study suggested that cardiomyocyte replication is a component of the cellular processes of ventricular remodelling. The potential occurrence of cardiomyocyte hyperplasia under special stimuli remains controversial. In the present study, the adult offspring rats from the LPS-exposed group showed myocardial remodelling, which was identified based on muscular and intramyocardial arterial components and the reduction of the cardiomyocyte nuclei profile number. We speculate that prenatal LPS exposure impaired the proliferative capacity of the cardiomyocytes in offspring rats.

In summary, the current study demonstrated that prenatal stress, such as exposure to a maternal inflammatory condition, is a key determinant of the development of myocardial remodelling, hypertension, and other dysfunctions in adult offspring. Furthermore, we believe that the NF-κB cascade is an important pathway in maternal inflammation-induced myocardial remodelling in offspring. Treatment processes that prevent maternal inflammatory responses would likely be useful in the prevention of cardiovascular and metabolic diseases, such as offspring myocardial remodelling and hypertension. A novel theory regarding the aetiology of offspring myocardial remodelling and hypertension may be derived from a clear delineation that myocardial remodelling and hypertension in adult offspring can result from pregnant mothers that are subjected to inflammatory stress.

## Abbreviations

LPS: Lipopolysaccharide; NF-κB: Nuclear transcription factor κB; PDTC: Pyrrolidine dithiocarbamate; LV: Left ventricular; BP: Blood pressure; CVD: Cardiovascular disease; CH: Cardiac hypertrophy; HF: Heart failure; TLR4: Toll-like receptor 4; SD: Sprague Dawley; LVESD: LV end-systolic diameter; LVEDD: LV end-diastolic diameter; PWT: Posterior wall thickness; IVST: Interventricular septum thickness; EF: Ejection fraction; FS: Fractional shortening; HI: Heart index; LVMI: LV mass index; TDM: Transverse diameter; CSA: Cross-sectional area; TUNEL: Transferase-mediated dUTP nick end labelling.

## Competing interests

The authors declare that they have no competing interests.

## Authors’ contributions

Y-l W participated in the design and implementation of the experiments and drafted the manuscript. W-h D and X-q X performed the echocardiographic evaluation. X-y H and P Y performed and analysed the animal experiments. Y-c D performed all of the WB experiments. X-h L and D-f C conceived the study, participated in its design and coordination, and drafted the manuscript. All authors read and approved the final manuscript.
